# Pervasive horizontal transmission of *Wolbachia* in natural populations of closely related and widespread tropical skipper butterflies

**DOI:** 10.1186/s12866-024-03719-1

**Published:** 2025-01-07

**Authors:** Pedro Ribeiro, Anzhelika Butenko, Daniel Linke, Hamid Reza Ghanavi, Joana Isabel Meier, Niklas Wahlberg, Pável Matos-Maraví

**Affiliations:** 1 Biology Centre of the Czech Academy of Sciences, Institute of Entomology, České Budějovice, Czech Republic; 2 Faculty of Science, University of South Bohemia, České Budějovice, Czech Republic; 3 Biology Centre of the Czech Academy of Sciences, Institute of Parasitology, České Budějovice, Czech Republic; 4 Life Science Research Centre, Faculty of Science, University of Ostrava, Ostrava, Czech Republic; 5 Functional Zoology Unit, Department of Biology, Lund University, Lund, Sweden; 6 Department of Zoology, University of Cambridge, Cambridge, UK; 7 Tree of Life Programme, Wellcome Sanger Institute, Wellcome Trust Genome Campus, Hinxton, Cambridge, UK; 8 Biodiversity Unit, Department of Biology, Lund University, Lund, Sweden

**Keywords:** Hesperiidae, *Wolbachia*, Phylogenetics, Population dynamics, Historical demography, Skipper butterflies, Double infection

## Abstract

**Background:**

The endosymbiotic relationship between *Wolbachia* bacteria and insects has been of interest for many years due to their diverse types of host reproductive phenotypic manipulation and potential role in the host’s evolutionary history and population dynamics. Even though infection rates are high in Lepidoptera and specifically in butterflies, and reproductive manipulation is present in these taxa, less attention has been given to understanding how *Wolbachia* is acquired and maintained in their natural populations, across and within species having continental geographical distributions.

**Results:**

We used whole genome sequencing data to investigate the phylogenetics, demographic history, and infection rate dynamics of *Wolbachia* in four species of the *Spicauda* genus of skipper butterflies (Lepidoptera: Hesperiidae), a taxon that presents sympatric and often syntopic distribution, with drastic variability in species abundance in the Neotropical region. We show that infection is maintained by high turnover rates driven mainly by pervasive horizontal transmissions, while also presenting novel cases of double infection by distantly related supergroups of *Wolbachia* in *S. simplicius.*

**Conclusions:**

Our results suggest that *Wolbachia* population dynamics is host species-specific, with genetic cohesiveness across wide geographical distributions. We demonstrate that low coverage whole genome sequencing data can be used for an exhaustive assessment of *Wolbachia* infection in natural populations of butterflies, as well as its dynamics in closely related host species. This ultimately leads to a better understanding of the endosymbiotic population dynamics of *Wolbachia* and its effects on the host’s biology and evolution.

**Supplementary Information:**

The online version contains supplementary material available at 10.1186/s12866-024-03719-1.

## Background

Bacteria of the genus *Wolbachia* stand as the cause of one of the largest and longest pandemics in Earth’s history with endosymbiotic relationships with arthropods and nematodes [1]. These Gram-negative bacteria are ubiquitous, present in approximately 20–30% of insect species in ecological communities [2, 3], and their actual incidence might encompass at least half of all arthropods on the planet [4]. The success of *Wolbachia* lies in its diverse interactions with hosts at the cellular level [5], which often lead to the manipulation of its hosts’ reproductive phenotypes. These include cytoplasmic incompatibility, feminization, parthenogenesis, and male killing [6–9]. *Wolbachia* also play a key role in the evolution of arthropods, as pervasive lateral gene transfer (LGT) from *Wolbachia* into host genomes might be responsible for chromosomal rearrangements or novel gene expression with beneficial effects on the host fitness [9–11]. There is, nonetheless, a profound gap in understanding how *Wolbachia* infection and distribution are maintained across and within species globally. Moreover, the evolutionary and biogeographical implications for host populations in widespread species remain unclear.

*Wolbachia* is acquired and spread across distinct host populations or species by three main mechanisms: (1) vertical transmission (cladogenetic), when diverging sister species acquire *Wolbachia* from a common ancestor [1, 12, 13]; (2) horizontal transmission among closely related host lineages, when *Wolbachia* infection establishes via reproductive exchange among populations of the same or different host species [13, 14]; and (3) horizontal transmission involving interactions with the environment (such as parasitoids or plants), implying that *Wolbachia* survives outside host cells for some time [1, 15–17]. The prevalence of *Wolbachia* is further modulated by the rate of infection loss within species [18], which may occur during the process of speciation [9, 19], caused by the acquisition of resistance by the host [20], or by displacement by another *Wolbachia* strain [21]. Distinguishing between these mechanisms and assessing their importance in maintaining the widespread distribution of *Wolbachia* requires studying their evolutionary history across populations of co-occurring, closely related host species [13, 14].

Lepidoptera (butterflies and moths) have emerged as a model host taxon in the study of *Wolbachia* endosymbiotic relationships [22]. The rates of infection in Lepidoptera species are among the highest in arthropods, and, contrary to other insect orders, Lepidoptera are predominantly infected with *Wolbachia* supergroup B rather than supergroup A [3] (*Wolbachia*’s major phylogenetic lineages are taxonomically divided into supergroups [23]). Although the infection rate at the species level can be high (e.g., up to ∼ 80% of all *Bicyclus* butterfly species; [24]), *Wolbachia* incidence at the population level varies from none to all individuals being infected [25, 26]. Further, Lepidoptera present the highest rate, among insects, of LGT from *Wolbachia* into their genomes, with important evolutionary implications, including the acquisition of genes with novel functions [10], population adaptation to the environment, sex determination, and the emergence of neo-W chromosomes [27]. Nonetheless, the study of the mechanisms involved in the acquisition and maintenance of *Wolbachia* in Lepidoptera hosts has received little attention from the perspective of infection gains and losses among closely related species whose populations overlap across biogeographical regions [25]. In this study, we aim to expand this knowledge by studying four butterfly species in a Neotropical genus (Hesperiidae: Eudaminae: *Spicauda*) that are sympatric and syntopic (i.e., they occur concomitantly in the same localities) in different areas of their widespread geographical distribution.

**Fig. 1:**
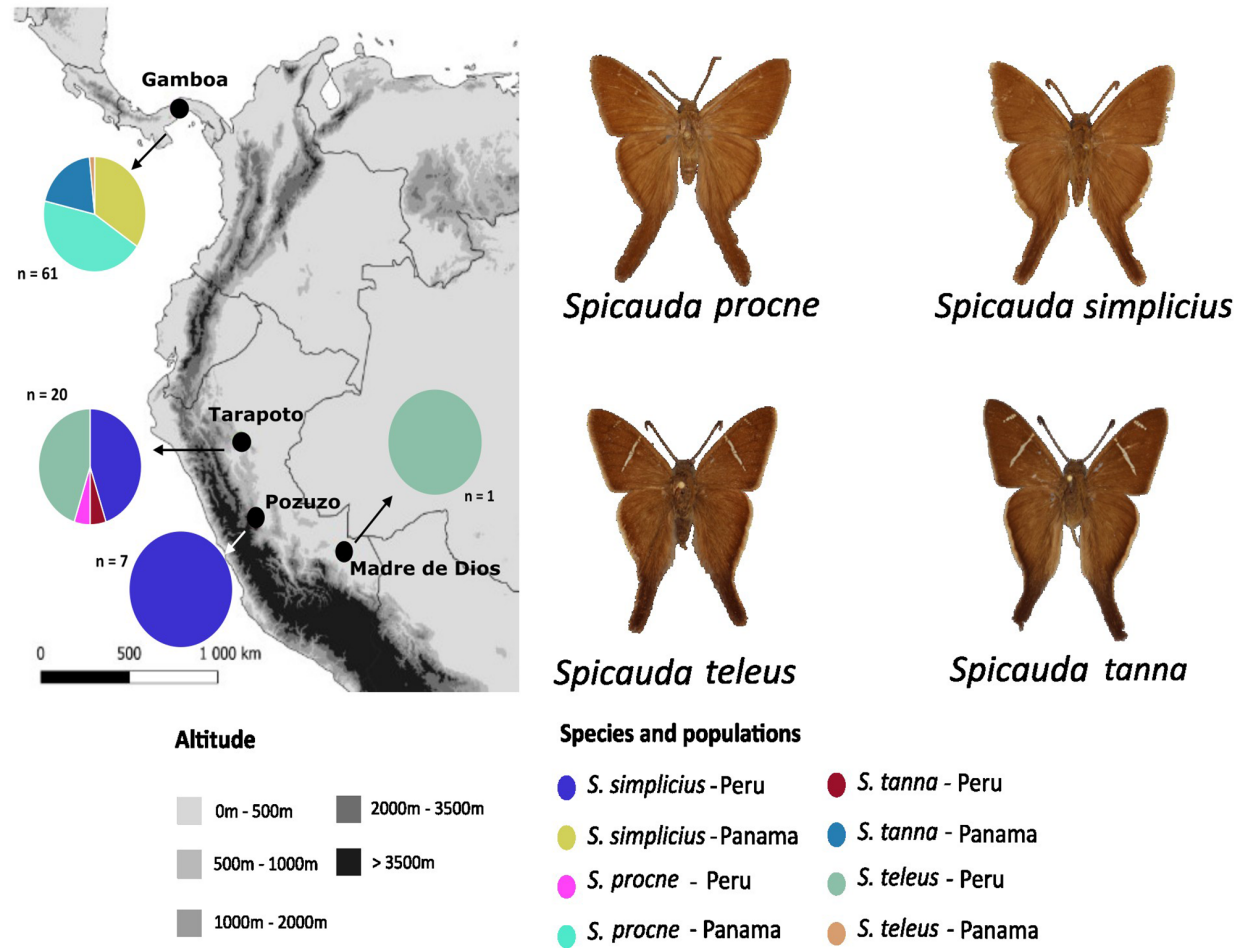
Sampling localities (left) and dorsal pictures of the focal species of this study (right). Map shows sampling localities in Peru (Tarapoto, Pozuzo, and Madre de Dios – east of the Andes) and Panama (Gamboa) along with pie charts indicating the proportion of species collected in each locality. The total number of specimens in each locality is shown next to each pie chart. Number of specimens per species is given in Methods section (see ‘Sample collection and DNA sequencing’)

*Spicauda* contains nine species with highly similar external adult morphologies: hindwing tails, brownish coloration, sometimes with white/transparent marks on the forewings [28, 29] (Fig. 1). While four species (*Spicauda simplicius*, *Spicauda procne*, *Spicauda tanna*, and *Spicauda teleus*) are widespread across the Neotropics, from southern USA to northern Argentina, local communities vary drastically in species abundance, and, in some areas, the four species are syntopic [29]. Such characteristics, together with findings that *Wolbachia* infect Eudaminae butterflies [22, 30], make *Spicauda* a suitable system to characterize the acquisition and spread of *Wolbachia* across biogeographical regions both between and within closely related species. Here, we use whole-genome resequencing data of specimens collected from populations east and west of the Andes (a major geographical barrier), in Peru and Panama respectively, to study infection rates, LGT from *Wolbachia* to the butterfly genomes, and the population dynamics histories of *Wolbachia* lineages in different host species.

## Methods

### Sample collection and DNA sequencing

We collected a total of 89 *Spicauda* specimens from four focal species (14 *S. tanna*, 10 *S. teleus*, 37 *S. simplicius*, and 28 *S. procne*). From these specimens, 28 were collected in Peru (east of the Andes, 20 from Tarapoto, seven from Pozuzo, and one from Madre de Dios) and 61 were collected in Panama (all from Gamboa) (Fig. 1, Additional File 1). Since the three localities in Peru are ecologically cohesive, we consider Peru and Panama as the only two distinct populations in our analyses. We used QIAGEN DNEasy Blood and Tissue DNA isolation kits, following the manufacturer’s protocol, to extract DNA from one to three entire legs per butterfly, based on the availability of tissue. The library preparation and whole-genome resequencing for all the Peruvian and part of the Panamanian samples were performed by Novogene (Cambridge, UK), and the remaining Panamanian samples were processed at Lund University, Sweden (library preparation following [31]) and sequenced by the National Genomics Infrastructure, NGI (Stockholm, Sweden). In both cases, the samples were sequenced using the Illumina NovaSeq 6000 system on a S4 v.1.5, PE 2 × 150 flowcell, aiming at ∼ 5Gb of data on average per butterfly sample. To generate a chromosome-level reference genome for this genus, another *S. simplicius* specimen collected in Pozuzo (Peru) was sequenced by the Wellcome Sanger Institute (UK) using HiFi (PacBio) and Hi-C (Illumina) technologies (NCBI accession: GCA_949699795.1). The laboratory protocols, chromosome-level genome assembly, and genome annotation are described in [32].

### Butterfly and *Wolbachia* genome assembly

We assessed the quality of Illumina reads before and after trimming with FastQC v.0.11.9 [33] and MultiQC v.1.20 [34]. We subsequently trimmed adapters and poly-G tails present in some samples using Fastp v.0.22.0 with default parameters [35]. We used SPAdes v.3.15.4 [36] to *de novo* assemble contigs of all samples using three different *k-mer* sizes: 21, 33, and 77. We used Quast v.5.2.0 [37] to assess basic assembly statistics. In this procedure, if any of our samples was infected by *Wolbachia*, then *Wolbachia* contigs would be assembled as a “by-product”.

Alternatively, we also assembled *Wolbachia* contigs using reads that exclusively mapped to *Wolbachia* genomes. To do this, we first used Bowtie2 v.2.4.2 [38] to map reads against *Wolbachia* reference genomes of supergroups A (NCBI accession: NC_002978.6) and B (NCBI accession: NC_010981.1), since they are the only two supergroups known to infect butterflies [22]. Then, we used SAMtools v1.14.0 [39] to convert SAM files to BAM, and to sort these BAM files into two files, one containing the reads that mapped to supergroup A and the other containing reads that mapped to supergroup B. We used BEDTools2 v.2.30.0 [40] and the command bamToFastq to convert each BAM file into FASTQ format. Finally, we used SPAdes v.3.15.4 [36] to assemble contigs for these sets of reads, resulting in *de novo* assembled *Wolbachia* contigs.

### Phylogenetic inference

We assessed the monophyly of *Spicauda* species and populations and investigated the evolutionary history of *Wolbachia* infecting our butterfly samples. We first extracted the protein-coding loci from the mitochondrial genomes of butterflies using MitoFinder v1.4.1 [41] with the default assembler MEGAHIT [42] and the mitogenome of *Cecropterus lyciades* (Hesperiidae: Eudaminae; NCBI accession: GCA_002930495.1) as a reference [43]. We used the MAFFT v.7.520 [44] plug-in in Geneious Prime 2023.2.1 (www.geneious.com; [45]) with the FFT-NS-i-x1000 algorithm, to obtain multispecies gene alignments, which were thoroughly inspected for errors and concatenated.

Second, we extracted protein-coding loci from the *de novo* assembled nuclear contigs of butterflies with the ‘*find_target_contigs*’ function in SECAPR v.2.2.3 [46, 47]. A set of 406 butterfly protein-coding loci from the target capture kit BUTTERFLY 1.0 [48] was used as a reference (Additional File 2). If multiple contigs matched a single reference locus, we selected the longest contig as potential homolog by flagging such contigs using the --*keep_paralogs* option. We then used MAFFT via the ‘*align_sequences*’ function in SECAPR to align the extracted contigs, and the --*keeplength* option in MAFFT to keep only the exonic regions of the reference loci (Additional File 2). We visually inspected the alignments for potential errors (misalignments, stop-codons, frame shifts) using Geneious, and included missing data in the alignments as “N”. Lastly, we concatenated the 406 alignments using the *catfasta2phyml.pl* script (available at https://github.com/nylander/catfasta2phyml.git).

To recover the phylogenetic relationships among the *Wolbachia* lineages infecting our butterfly samples, we used two datasets: a set of five legacy multilocus sequence typing genes (MLST; housekeeping genes *gatB*, *coxA*, *hcpA*, *ftsZ*, and *fbpA*) plus the *wsp* gene used for *Wolbachia* screening, and a set of 210 loci to improve the phylogenetic resolution. For the first dataset, we followed the SECAPR procedure described above to extract the protein-coding MLST and *wsp* loci, from the *de novo* contigs assembled by SPAdes using the mapped reads to *Wolbachia* reference genomes. As references for the extraction of such loci, we used different *Wolbachia* supergroups (A, B, D, and F), including the strains *w*Bm, *w*No, wPel, *w*Au, *w*Ha, *w*MelPop, *w*Ri, *w*Irr, *w*Clec-F, *w*Vit, *w*Stri, and *w*Bol1, from the alignments available in [30]. For the second dataset, we used the set of 210 protein-coding loci available in [49] as references. The process of extraction of contigs was the same as described above. Due to the potential presence of paralogs in this phylogenomic dataset, we selected only those alignments for which average pairwise identity was higher than 89% (i.e., the percentage of pairwise residues that are identical in the alignment, including gap versus non-gap residues, but excluding gap versus gap residues, as calculated in Geneious). All vetted alignments are available in the Zenodo repository (https://zenodo.org/records/12703938; Additional File 2).

Finally, we used the maximum likelihood approach implemented in IQ-TREE 2 v.2.3.5 [50] to infer phylogenetic trees from butterfly nuclear and mitochondrial genes, and from the two sets of *Wolbachia* loci. For each of the four concatenated alignments, we first used PartitionFinder2 v.2.1.1 [51] to assess the best partition scheme, with the greedy search algorithm [52], and the RAxML option [53]. In IQ-TREE 2, we used 1,000 ultrafast bootstrap iterations [54] with the *alrt* correction, *bnni* optimization, and extended model selection (*-m* MFP option, selected models can be found in Zenodo (Additional File 2). We also ran IQ-TREE 2 for each of the six MLST *Wolbachia* loci separately to assess their contribution to the concatenated *Wolbachia* phylogeny using the same parameters as before.

### Confirmation of *Wolbachia* infections

We used a series of confirmation steps to corroborate the results of the extraction of MLST and *wsp* from our samples. First, we used BLAST v.2.10.0 [55] with the BLASTX option to search for *Wolbahcia* proteins on the *de novo* contigs assembled from the total read data of each butterfly sample. We searched the assembled contigs against a database of annotated proteins of *Wolbachia* from supergroups A (NCBI accession: NC_002978.6) and B (NCBI accession: NC_010981.1). The rationale of using BLASTX was to accurately identify *Wolbachia* contigs that might have been assembled from the butterfly samples data, using conservative filters, i.e., percentage identity > 95%, bitscore > 300, and *e*-value < 1×*e*^-10^. The samples that passed these filtering criteria were considered infected by *Wolbachia*, and the contigs identified as *Wolbachia* were stored in new FASTA files.

Then, we ran a BUSCO v.5.5.0 search [56] using the set of identified *Wolbachia* contigs to assess their completeness with the bacteria_odb10 as the reference dataset. In addition, we followed the procedure proposed in [57] to assess the evenness of coverage of two *Wolbachia* reference genomes by our sequencing reads. For this, we first mapped all the reads using Bowtie2 v.2.4.2 [38] with the ‘*very sensitive local*’ algorithm, against a reference of combined *Wolbachia* genomes belonging to supergroups A and B (same NCBI accessions as used for the *Wolbacia* genome assemblies). We used SAMtools v.1.14 [39] to convert SAM files to BAM and to sort the resulting BAM files. Then, we extracted only the mapped reads using the bamToFastq command in BEDTools v.2.30.0 [40] for a subsequent re-mapping step (using Bowtie2 v.2.4.2), with the same combined *Wolbachia* genomes reference. Finally, we used SAMtools v.1.14 [39] to calculate the evenness of coverage of the *Wolbachia* reference genomes.

### Demographic history of *Wolbachia* infecting *Spicauda*

We inferred the effective population size dynamics of the *Wolbachia* supergroups infecting *S. simplicius* and *S. procne* using the Bayesian skyline plot (BSP) method [58] implemented in BEAST v.2.7.6 [59]. We concatenated the MLST and *wsp* genes into three datasets, i.e., supergroups A and B infecting *S. simplicius*, and supergroup B infecting *S. procne*. Because the BSP method assumes the population is panmictic [60], we removed the phylogenetically highly divergent sample BC007 (*Wolbachia *supergroup B infecting *S. simplicius*) from the analyses. For each dataset, we used the substitution model estimated by Bayesian information criterion values in jModelTest v.2.1.10 [61], a single strict molecular clock, and a single tree model with five groups of coalescent intervals and the piecewise-constant skyline analysis. We ran four independent analyses for each dataset, consisting of 100 million generations and sampling frequency of 10,000. We applied a burn-in of 25% and combined the posterior estimates of the four independent analyses using LogCombiner (part of the BEAST2 package). In Tracer v.1.7.1 [62], we confirmed convergence of independent runs and that the effective sample sizes (ESS) were above 200, before performing a Bayesian Skyline analysis under the stepwise (constant) model.

### Potential lateral gene transfers from *Wolbachia* into *S*. *simplicius*

Since we detected a presumably pseudogenized *Wolbachia fbpA* gene (as judged by the presence of in-frame stop codons - Additional File 2) in some *S. simplicius* specimens, we screened the chromosome-level genome assembly of *Spicauda simplicius* [32] for signatures of LGTs from *Wolbachia*. For this, we used BLAST v.2.10.0 [55] for the coding sequences (CDS) from *Wolbachia* supergroup B (*w*Pip; NC_010981) as BLASTN queries, because our phylogenetic analyses revealed that the *fbpA* pseudogenes clustered within *Wolbachia* supergroup B lineages. The reference genome assembly of *S. simplicius* [31] was used as the BLASTN database. Because genes originating through LGT might be to some extent degraded, we relaxed our BLASTN filtering criteria to incorporate the matches having percentage identity higher than 90%. Additionally, we ran IQ-TREE 2 exclusively for the *fbpA* gene, but in this case including the specimens containing the presumably pseudogenized sequences, although keeping only the conserved part of these sequences (Additional File 2).

## Results

### Butterfly and *Wolbachia* genome assembly

Our sequencing of the butterfly samples resulted in high quality reads and an average of ∼ 11 Gb of data per sample (read quality statistics can be found in Zenodo; Additional File 2). The genome assembly process resulted in an average of 295,243 *de novo* contigs per sample, with an average N_50_ of 1,979 bp (Additional File 3). We also retrieved *Wolbachia* contigs after mapping the reads against *Wolbachia* reference genomes for the butterfly species *S. simplicius* and *S. procne*, but not for *S. tanna* and *S. teleus*. The average total length of assemblies for single-infected specimens (i.e., reads only mapped to one reference genome) was 1,144,358 bp, with the largest contig being 129,752 bp long (average contig size, 55,215 bp) (Additional File 3). Twelve specimens of *S. simplicius* were potentially double-infected (i.e., reads mapped to both reference genomes) and presented an average total assembly length of 473,803 bp, and contigs were smaller than those from single-infected specimens (largest contig = 27,727 bp, average contig size, 10,043 bp) (Additional File 3).

### Phylogenetic inference

**Fig. 2:**
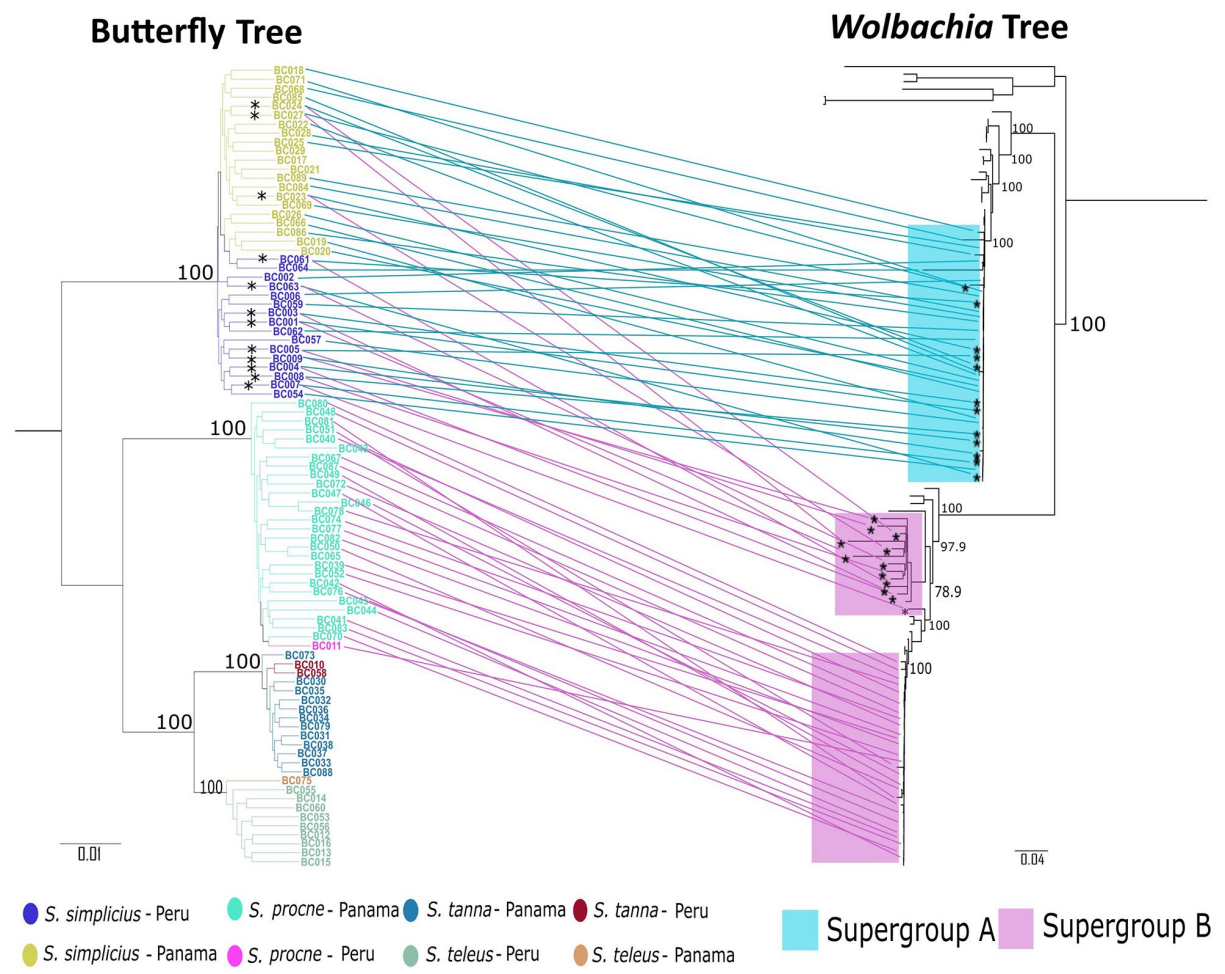
Phylogenetic trees inferred with IQ-TREE 2 using sets of butterfly nuclear (left) and *Wolbachia* (right) loci. The colors at the tips of the butterfly nuclear tree (406 protein coding loci) represent the species and the specimen’s population of origin (Peru or Panama), and only the branches supported by ultrafast bootstrap value of 100 are shown. The *Wolbachia* tree (108 protein coding loci) shows that *S. procne* is mainly infected by *Wolbachia* supergroup B while *S. simplicius* is mostly infected by supergroup A. Some specimens of *S. simplicius* are also infected by *Wolbachia* supergroup B. The colored rectangles highlight the two distinct *Wolbachia* supergroups infecting the studied specimens. We show only the ultrafast bootstrap support for the relationships among the supergroups infecting our specimens. Asterisks identify double-infected individuals of *S. simplicius* and the colored lines in the tanglegram represent the different *Wolbachia* supergroups infecting our studied butterflies. Both tree files with complete information can be found in the Zenodo repository (Additional File 2)

We retrieved an average of 379 butterfly nuclear genes per sample from the BUTTERFLY 1.0 reference of 406 protein coding loci (average missing data of approximately 6.65% of all target genes), and the concatenated butterfly alignment was 236,864 bp long. For the mitochondrial genome, we recovered all *Spicauda* mitochondrial protein-coding genes, except for *ATP8*, which was not identified in any of the samples. The concatenated mitochondrial alignment consisted of 12 coding loci containing 10,911 bp. Both nuclear and mitochondrial butterfly datasets supported the same tree topology, in which *S. tanna* and *S. teleus* are sister species, *S. procne* is sister to both, and *S. simplicius* is sister to all of them (Fig. 2 - nuclear tree; Additional File 4 Fig. S1 - mitochondrial tree). All species were monophyletic regardless of its population of origin.

Since the MLST and *wsp* markers are commonly used to identify *Wolbachia* infection, we used these markers as a first line of identification of the supergroups infecting our samples. The rationale is that if a specimen was infected, we would be able to extract most of the MLST and *wsp* loci from *Wolbachia* contigs assembled along the butterfly contigs. We found that 33 out of 37 (∼ 89.2%) *S. simplicius* specimens, and all 28 *S. procne* specimens were infected with *Wolbachia* (Additional File 5). None of the *S. tanna* and *S. teleus* were infected, as also suggested by the lack of *Wolbachia* reads after mapping to reference *Wolbachia* genomes.

After applying a restrictive pairwise identity filter to the 210 *Wolbachia* loci, we obtained a total of 108 genes that were used for a phylogenomic analysis. We corroborated our findings based on the MLST and *wsp* loci, in that the *Wolbachia* identified in *S. procne* clustered within the *Wolbachia* supergroup B, while those from *S. simplicius* were closely related to the supergroup A (Fig. 2). Moreover, our phylogenetic analyses showed that 12 *S. simplicius* specimens contained markers of both *Wolbachia* supergroups A and B, suggesting a double infection in those individuals. The same 12 specimens contained sufficient number of reads to assemble both supergroup A and supergroup B contigs. This analysis also showed that the *Wolbachia* supergroup B infecting *S. simplicius* and *S. procne* belonged to two highly divergent lineages (Fig. 2). Lastly, the only disagreement with the phylogenetic tree obtained with the MLST and *wsp* loci (Additional File 6 Fig. S2), was the position of the supergroup B *Wolbachia* lineage found in the sample BC007, a double-infected *S. simplicius.* This lineage is highly divergent from other *Wolbachia* found in double-infected *S. simplicius* specimens. All phylogenetic tree files can be found in the Zenodo repository (Additional File 2).

### Confirmation of *Wolbachia* infection

The genome-wide data facilitated the identification of contigs that contained genes from *Wolbachia* supergroups A and B. The results of the BLASTX search using a strict filter in terms of bitscore, percentage identity and *e*-value against *Wolbachia* proteins confirmed the results obtained with the MLST markers, in which 33 out 37 (∼ 89.2%) *S. simplicius* and all 28 specimens of *S. procne* were infected, whilst *S. tanna* and *S. teleus* were not, regardless of the sampled population. All BLASTX results in TSV files (before filtering) are provided in Zenodo (Additional File 2).

The BUSCO analysis against the bacterial reference database showed high levels of completeness (on average, ∼ 74% - Additional File 7) of the identified *Wolbachia* contigs, which were also evenly covered with sequencing reads (Additional File 8). The samples BC019, BC020 (both from *S. simplicius*), BC043, BC044, BC045, and BC046 (from *S. procne*), even though identified as infected, presented a much lower percentage of complete BUSCOs, with an average of 13.31%. This might be explained by the lower number of raw reads in these samples compared to the others (Additional File 1), which was also reflected in the lower read coverage in *Wolbachia* genomes (Additional File 8).

### Historical population dynamics of *Wolbachia* infecting *S. procne* and *S. simplicius*

**Fig. 3:**
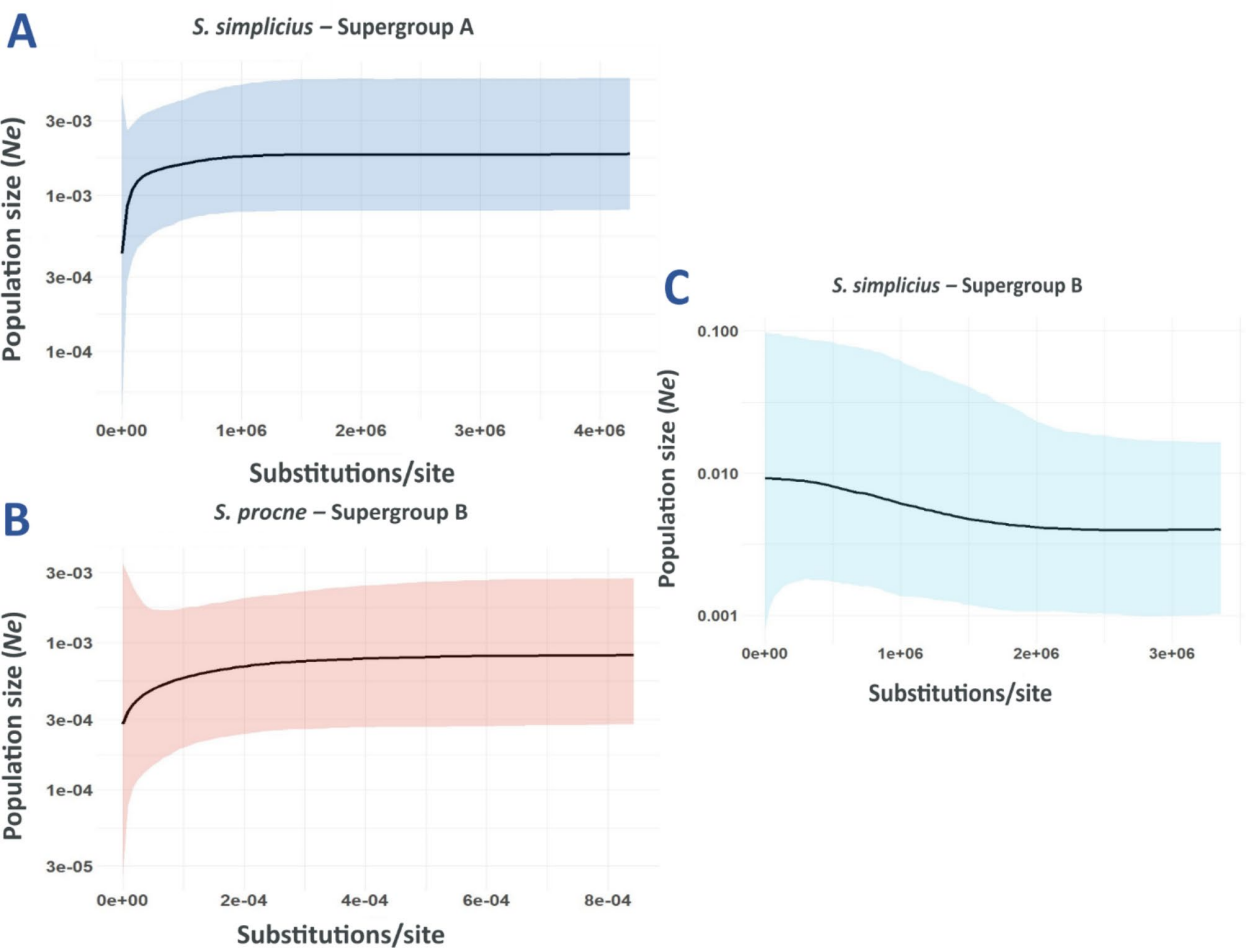
Bayesian skyline plots showing the historical demography of *Wolbachia* supergroups A and B infecting *Spicauda.* **A.** Recent contraction of *Wolbachia* supergroup A infecting most of *S. simplicius* specimens. **B. ***Wolbachia* supergroup B infecting *S. procne* shows a mostly constant *Ne* through time with a sign of contraction towards the present. **C.** Recent expansion of *Wolbachia* supergroup B infecting 12 specimens of *S. simplicius.* BC007 specimen was excluded due to a high divergence from other of the *Wolbachia* supergroup B infecting *S. simplicius*

The *Wolbachia* supergroup A infecting *S. simplicius* and the supergroup B infecting *S. procne* likely had distinct, species-specific population dynamics. While the *Wolbachia* supergroup A from *S. simplicius* had a recent contraction in effective population size (*Ne*), *Wolbachia* supergroup B infecting *S. procne* presented an approximately constant population size through time. Moreover, the supergroup B lineage that also infected the 12 specimens of *S. simplicius*, showed a demographic history of gradual expansion, which contrasts with the supergroup B lineage infecting *S. procne*. The *Ne* of the supergroup B infection in *S. simplicius* is approximately 20-fold higher than that of the supergroup B in *S. procne* and supergroup A in *S. simplicius* (Fig. 3). These two latter infections had similar *Ne* in the present.

### Potential lateral gene transfer from *Wolbachia* to *Spicaudasimplicius*

**Fig. 4:**
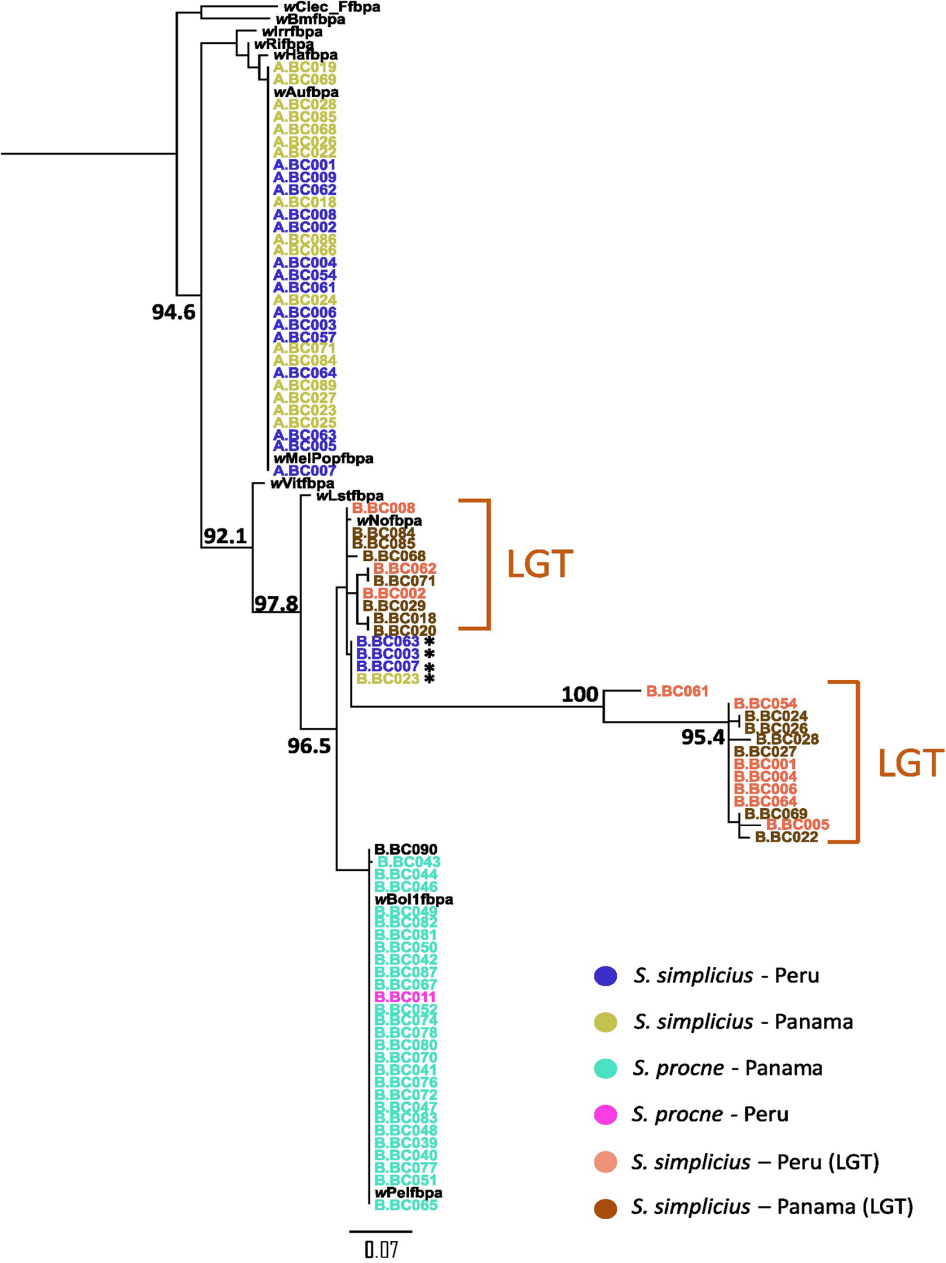
Phylogenetic tree based on *Wolbachia* *fbpA* gene sequences. The tree highlights 23 specimens of *S. simplicius* that present in-frame stop codons in the *fbpA* gene (LGT, potential lateral gene transfer into *S. simplicius* nuclear genome). These specimens are more closely related to the supergroup B infecting other four *S. simplicius* specimens that possess a complete *fbpA* gene (marked with an asterisk in the figure), instead of supergroup B infecting *S. procne*. We used IQ-TREE 2, and only ultrafast bootstrap support values over 90 and on nodes highlighting the relationships among the *Wolbachia* supergroups infecting our specimens are shown

We found CDS regions of *Wolbachia* supergroup B that matched regions in the chromosome-level assembly of *S. simplicius* (BLASTN percentage identity > 90% and query coverage > 10%). These included *Wolbachia*’s thioredoxin in *S. simplicius* chromosome 1, phage terminase large subunit in *S. simplicius* chromosome 7, fructose-bisphosphate aldolase (*fpbA*) in *S. simplicius* chromosome 9, a hypothetical *Wolbachia* protein in *S. simplicius* chromosome 12, and phage baseplate assembly protein V in *S. simplicius* chromosome 26. One of these putative *Wolbachia* genes identified in *S. simplicius* chromosomes was the MLST marker *fbpA*, which was consistently found containing in-frame stop codons in 23 *S. simplicius* samples (Fig. 4 – marked as LGT). The presumably pseudogenized *fbpA* sequence in these samples was phylogenetically closely related to the *Wolbachia*-derived *fbpA* sequence present in some double-infected *S. simplicius* specimens (Fig. 4). Some of these specimens form a clade that has, according to branch lengths, accumulated more mutations over time than other *Wolbachia* supergroup B infecting both *S. simplicius* and *S. procne* (Fig. 4).

## Discussion

We assessed the evolutionary history and population dynamics of *Wolbachia* infecting four closely related species of the butterfly genus *Spicauda*, a typically syntopic and geographically widely distributed genus in the Neotropics. By using a battery of approaches, from genome assembly to phylogenetic inference, we identified butterfly species infected by different *Wolbachia* supergroups. We found that two butterfly species, *S. procne* and *S. simplicius*, carried species-specific and divergent lineages of *Wolbachia* belonging to supergroups A and B, regardless of their syntopic occurrence and phylogenetic relatedness. Moreover, since specimens from the same species were mainly infected with the same *Wolbachia* supergroup, regardless of their population of origin (Peru or Panama), we propose that infection is cohesive and widespread, even across important geographical barriers like the Andes. Since *S. tanna* and *S. teleus*, and their extant populations in Peru and Panama were not infected by *Wolbachia*, we propose that the most recent common ancestor of both sister butterfly species was likely *Wolbachia-*free. Alternatively, any infection in the past in such butterfly lineages was lost, reinforcing the idea of high turnover rates.

Our results suggested that cladogenetic, vertical transmission among species [15] is an unlikely scenario because of the high divergence of *Wolbachia* supergroups infecting *S. simplicius* and *S. procne*. Moreover, 12 specimens of Peruvian and Panamanian *S. simplicius* have been simultaneously infected by lineages from *Wolbachia* supergroups A and B, which show contrasting demographic history patterns: on one hand, supergroup A infecting these specimens showed recent signs of contraction of effective population size, whereas supergroup B showed recent expansion. Thus, we propose that such divergence of infecting *Wolbachia* supergroups and their distinct infection demographic histories make rampant horizontal transmission the main driving mechanism of *Wolbachia* acquisition and spread in these closely related butterfly species.

### *Wolbachia* infection dynamics in *Spicauda*: acquisition, rates of infection, and phylogenetic history

High rates of *Wolbachia* infection are common in butterfly species [3, 24], whilst at the population level, *Wolbachia* incidence can vary drastically [25]. In *Spicauda*, we found high variation, in which more than 90% of *S. procne* and *S. simplicius* specimens were infected, whilst *S. tanna* and *S. teleus* appeared to be *Wolbachia*-free. Importantly, despite potential low-titer systemic infections in our butterfly samples, a false negative scenario in *S. tanna* and *S. teleus* is unlikely, since sampling effort and laboratory protocols (i.e., DNA extracted from one to three legs and short-read sequencing) were comparable for all syntopic *Spicauda* species in every population. However, studies focusing on other populations across the Neotropics are necessary to confirm that the lack of *Wolbachia* infections is species-specific. If such a pattern holds, either the loss of *Wolbachia* or the resistance to the infection might have occurred in the most recent common ancestor of *S. tanna* and *S. teleus*.

On the contrary, *Wolbachia* is highly prevalent (100%) in the Panamanian population of *S. procne*. Even though our sampling was exhaustive and reflects local butterfly abundances, we were able to collect only one specimen of *S. procne* in Peru, and this specimen was also infected by the supergroup B lineage characteristic for the samples from Panama (Fig. 2). *Spicauda simplicius*, the most abundant species in both populations, showed a high rate of *Wolbachia* infection, with 93.8% of specimens from Peru and 88.2% from Panama. Almost all specimens in both populations are infected by a single supergroup A lineage of *Wolbachia*, indicating that the infection is genetically cohesive despite the wide geographical distribution of the host. Nonetheless, 12 (32.4%) specimens of *S. simplicius* (9 from Peru and 3 from Panama), were identified as simultaneously infected by at least two divergent lineages of *Wolbachia* supergroup B (Fig. 2), which were different from the strains infecting *S. procne*. Multiple *Wolbachia* infection by different strains of the same supergroup has been reported in butterflies [63], and even up to five different strains in the same individual have been reported in other insects [64]. On the other hand, double infection by two distinct supergroups, as reported here for *S. simplicius*, is a much rarer phenomenon. For instance, out of 93 species analyzed with chromosome-level genomes in [3], only 5 (∼ 5.4%) presented infection by supergroups A and B concomitantly, including only two Lepidoptera species. In addition to identifying new cases of double infection in Lepidoptera, we show that each infecting *Wolbachia* supergroup can have distinct population dynamics and evolutionary histories. Hence, it is possible that this phenomenon is an important part of the acquisition, spread, and turnover of *Wolbachia* in naturally occurring populations.

Our phylogenetic analyses showed that despite the syntopic occurrence and phylogenetic relatedness of host species, *Wolbachia* infection in *Spicauda* is species-specific. The supergroup B strain infecting *S. procne* is distantly related to the supergroup B infecting *S. simplicius* (Fig. 2). In fact, supergroup B infecting *S. procne* is more closely related to the *Wolbachia* supergroup B strain infecting *Cecropterus dorantes* (sample BC090), which is a more distantly related Eudaminae butterfly species, than to the supergroup B infecting *S. simplicius* (Fig. 2; Additional File 6 Fig. S2). In natural populations of the closely related butterflies *Lycaeides melissa* and *L. idas*, *Wolbachia* was acquired via horizontal transmission facilitated by introgression even when sequence differences in the infecting strains are small (i.e., one identified mutation) [25]. Thus, the presence of distantly related strains of supergroup B infecting *S. procne* and some specimens of *S. simplicius* aligns with the endosymbiont’s acquisition via horizontal transmission between unrelated populations, or by hybridization between different closely related species.

Lastly, it has been shown that phylogenies based only on a handful of genes, such as the MLST and *wsp* markers, might be less accurate and sometimes discordant with phylogenomic datasets [65, 66]. On the other hand, our results showed that both datasets revealed the same phylogenetic tree topology for *Wolbachia*, in agreement with [49]. Since obtaining genomic loci is not feasible in every study, using only the MLST and *wsp* markers is sufficient for *Wolbachia* screening and for gaining the first insight into their evolutionary history in host’s populations.

### Effective population size dynamics of *Wolbachia* infection in *Spicauda*

With the demographic history analysis of *Wolbachia*, we shed light on how the extant prevalence across populations is maintained. The population dynamics history of *Wolbachia* in *S. procne* is explained by a constant *Ne* through time (Fig. 3). However, the supergroup B lineage infecting *S. simplicius* seems to be a more recent expanding infection than supergroup A in *S. simplicius*, which has signatures of recent contraction in effective population size (*Ne*) after a nearly constant size through time (Fig. 3). This demographic pattern has been shown in other butterflies and in *Drosophila*, wherein infection loss has been driven by a replacement with a distinct *Wolbachia* strain [15, 25]. Confirming this *Wolbachia* replacement hypothesis of supergroup A by supergroup B in *S. simplicius*, however, would require further investigation such as estimating competition between supergroups co-infecting the same host [65], by assessing the titer infection in double-infected individuals. In our case, this procedure is not feasible due to the varied nature of our sequencing approaches, including varying amounts of tissue per sample and performing library preparations and Illumina sequencing at different facilities. Our results, nonetheless, clearly show a more recent infection of supergroup B in double-infected *S. simplicius* specimens, with an expansion in *Ne.* Therefore, we propose that the prevalence and maintenance of *Wolbachia* in closely related species might be driven by high turnover of new strains and supergroups of *Wolbachia*, leading to potentially fast strain replacements or increase in rates of co-infection by different supergroups.

Lastly, the phylogenetic divergences of the supergroup B strains infecting *S. simplicius* and *S. procne* are shallow within species, suggesting that the infection is widespread and phylogenetically cohesive between Peru and Panama. However, it is important to note that as haplotype phasing using short Illumina reads is challenging, we were not able to detect any closely related strains within the same *Wolbachia* supergroups, thus leading to a potential underestimation of *Wolbachia* diversity in *S. simplicius* and *S. procne* [3, 30].

### Putative lateral gene transfer of *Wolbachia* genes to *S*. *simplicius*’ genome

Genomic data has allowed for a more robust identification of LGT between a prokaryotic donor and a eukaryotic host [67], and it has been shown that the process is widespread in insects. Recently, 1,410 laterally transferred genes have been found in 192 insect species, of which 533 genes originated from bacteria [10]. *Wolbachia* is a major donor in this scenario and, more importantly, Lepidoptera is the insect order in which many cases of LGT have been reported with an average of 16 genes per analyzed species [10]. Nonetheless, accounting for *Wolbachia* LGT into host genome is not trivial. We assessed the extent of LGT from *Wolbachia* to the genome of *S. simplicius* using homology-based searches to identify fragments of bacterial genes in the chromosome-level reference genome assembly of the butterfly host. One of the identified fragments corresponds to the gene encoding fructose bisphosphate-aldolase (*fbpA*), an important enzyme involved in carbohydrate metabolism. The phylogenetic placement of such a fragment presumably transferred to *S. simplicius*, suggests that its origin is traced back to *Wolbachia* that was closely related to the extant supergroup B lineages infecting *Spicauda* (Fig. 4). Additionally, some of the putative laterally transferred sequences formed a clade that had a long stem branch, which could be indicative of either gene erosion or higher substitution rates due to loss of functionality in the genome of *S. simplicius* [11] (Fig. 4). Taken together, this suggests that *S. simplicius* might have been ancestrally infected by a supergroup B lineage, which was likely distinct from the extant infection in *S. procne.* Future studies should focus on identifying LGT more thoroughly across populations, which was not possible here due to low sequencing coverage and the lack of reference genomes for the other *Spicauda* species. Such studies would shed light on the role of LGT from endosymbionts in shaping the evolution of their hosts.

## Conclusions

*Wolbachia* infections of insects are one of the most important symbiotic relationships between bacteria and eukaryotes. Its prevalence in butterfly species as well as its diverse acquisition and spread dynamics at the population level have been thoroughly assessed. Moreover, it is known that reproductive manipulations by *Wolbachia* are common in butterflies [22] with potential implications for host’s evolution [11]. The availability of genomic data for Lepidopetera [3, 68, 69], and vast literature on their ecology and distribution makes this order a prominent system to further investigate *Wolbachia* infection dynamics in closely related species with wide geographical distribution. We show that low-coverage whole genome sequencing data enable the accurate identification of *Wolbachia* infection in insect specimens. Moreover, we show that *Wolbachia* infections in extant natural populations of closely related butterfly species are likely maintained by high turnover rates driven mainly by pervasive horizontal transmissions. Our results indicate that the loss of infection is likely to occur in closely related species regardless of their sympatric and syntopic occurrence as is the case for *S. tanna* and *S. teleus.* Also, the *Wolbachia* infections we uncovered in *S. simplicius* and *S. procne* seem genetically cohesive within each species, even in populations across important geographical barriers like the Andes. This may partially be explained by the high dispersal capabilities of skipper butterflies [70]. Finally, we show that investigating *Wolbachia’s* phylogenetic history and its demographic dynamics is necessary to understand its pervasive acquisition mechanisms in natural populations and shed light on how it spreads regardless of relatedness, distribution, and occurrence of host species.

## Electronic supplementary material

Below is the link to the electronic supplementary material.

Supplementary Material 1

Supplementary Material 2

Supplementary Material 3

Supplementary Material 4

Supplementary Material 5

Supplementary Material 6

Supplementary Material 7

Supplementary Material 8

## Data Availability

The raw data of the samples used in this study are already assigned to BioProject PRJNA1085524. All Short Read Archive (SRA) data are published and available for download in NCBI. The respective SRR Accession numbers for all samples are provided in Additional File 1.xlsx. All alignments used in our analyses, TSV files obtained for the BLASTX results, evolutionary models used in analyses, tree files obtained with IQTree 2, and HTML FastQC results are already published with an embargo in Zenodo. The link for this Zenodo publication is https://zenodo.org/records/12703938 and files are publicly available.

## References

[1] Gerth M, Röthe J, Bleidorn C. Tracing horizontal *Wolbachia* movements among bees (Anthophila): a combined approach using multilocus sequence typing data and host phylogeny. Mol Ecol. 2013;22(24):6149–62. 24118435 10.1111/mec.12549

[2] West SA, Cook JM, Werren JH, Godfray HCJ. *Wolbachia* in two insect host-parasitoid communities. Mol Ecol. 1998;7(11):1457–65. 9819901 10.1046/j.1365-294x.1998.00467.x

[3] Vancaester E, Blaxter M. Phylogenomic analysis of *Wolbachia* genomes from the Darwin Tree of Life biodiversity genomics project. PLoS Biol. 2023;21(1):e3001972. 10.1371/journal.pbio.3001972 PMC9894559 36689552

[4] Weinert LA, Araujo-Jnr EV, Ahmed MZ, Welch JJ. The incidence of bacterial endosymbionts in terrestrial arthropods. Proc R Soc B Biol Sci. 2015;282(1807):3–8. 10.1098/rspb.2015.0249 PMC4424649 25904667

[5] Porter J, Sullivan W. The cellular lives of *Wolbachia*. Nat Rev Microbiol. 2023;21:750–66. 10.1038/s41579-023-00918-x 37430172

[6] Hoffmann AA, Turelli M, Simmons GM. Unidirectional incompatibility between populations of *Drosophila simulans*. Evolution. 1986;40(4):692–701. 10.1111/j.1558-5646.1986.tb00531.x 28556160

[7] Hurst GDD, Jiggins FM. Male-killing bacteria in insects: mechanisms, incidence, and implications. Emerg Infect Dis. 2000;6(4):329–36. 10905965 10.3201/eid0604.000402 PMC2640894

[8] Stouthamer R. *Wolbachia*-induced parthenogenesis. In: O’Neill SL, Hoffmann AA, Werren JH, editors. Influential passengers: inherited microorganisms and arthropod reproduction. Oxford University Press; 1997. pp. 102–24.

[9] Werren JH, Baldo L, Clark ME. *Wolbachia*: master manipulators of invertebrate biology. Nat Rev Microbiol. 2008;6(10):741–51. 10.1038/nrmicro1969 18794912

[10] Li Y, Liu Z, Liu C, Shi Z, Pang L, Chen C, et al. HGT is widespread in insects and contributes to male courtship in lepidopterans. Cell. 2022;185(16):2975–87.e10. 10.1016/j.cell.2022.06.014 PMC9357157 35853453

[11] Nikoh N, Tanaka K, Shibata F, Kondo N, Hizume M, Shimada M, et al. *Wolbachia* genome integrated in an insect chromosome: evolution and fate of laterally transferred endosymbiont genes. Genome Res. 2008;18(2):272–80. 10.1101/gr.7144908 PMC2203625 18073380

[12] Bandi C, Anderson TJC, Genchi C, Blaxter ML. Phylogeny of *Wolbachia* in filarial nematodes. Proc R Soc B Biol Sci. 1998;265(1413):2407–13. 10.1098/rspb.1998.0591 PMC1689538 9921679

[13] Raychoudhury R, Baldo L, Oliveira DCSG, Werren JH. Modes of acquisition of *Wolbachia*: horizontal transfer, hybrid introgression, and codivergence in the *Nasonia* species complex. Evolution. 2009;63(1):165–83. 10.1111/j.1558-5646.2008.00533.x 18826448

[14] Turelli M, Cooper BS, Richardson KM, Ginsberg PS, Peckenpaugh B, Antelope CX, et al. Rapid global spread of *w*Ri-like *Wolbachia* across multiple *Drosophila*. Curr Biol. 2018;28(6):963–71.e8. 10.1016/j.cub.2018.02.015 PMC5882237 29526588

[15] Cooper BS, Vanderpool D, Conner WR, Matute DR, Turelli M. *Wolbachia* acquisition by *Drosophila yakuba*-clade hosts and transfer of incompatibility loci between distantly related *Wolbachia*. Genetics. 2019;212(4):1399–419. 10.1534/genetics.119.302349 PMC6707468 31227544

[16] Huigens ME, De Almeida RP, Boons PAH, Luck RF, Stouthamer R. Natural interspecific and intraspecific horizontal transfer of parthenogenesis-inducing *Wolbachia* in *Trichogramma* wasps. Proc R Soc B Biol Sci. 2004;271(1538):509–15. 10.1098/rspb.2003.2640 PMC1691627 15129961

[17] Turelli M, Hoffmann AA. Cytoplasmic incompatibility in *Drosophila simulans*: dynamics and parameter estimates from natural populations. Genetics. 1995;140(4):1319–38. 10.1093/genetics/140.4.1319 PMC1206697 7498773

[18] Bailly-Bechet M, Martins-Simões P, Szöllosi GJ, Mialdea G, Sagot MF, Charlat S. How long does *Wolbachia* remain on board? Mol Biol Evol. 2017;34(5):1183–93. 10.1093/molbev/msx073 28201740

[19] Baldo L, Hotopp JCD, Jolley KA, Bordernstein SR, Biber SR, Raychoudhury R, et al. Multilocus sequence typing system for the endosymbiont *Wolbachia pipientis*. Appl Environ Microbiol. 2006;72(11):7098–110. 10.1128/AEM.00731-06 PMC1636189 16936055

[20] Correa CC, Ballard JWO. *Wolbachia* associations with insects: winning or losing against a master manipulator. Front Ecol Evol. 2016;3:153.

[21] Kriesner P, Hoffmann AA, Lee SF, Turelli M, Weeks AR. Rapid sequential spread of two *Wolbachia* variants in *Drosophila simulans*. PLoS Pathog. 2013;9(9):e1003607. 10.1371/journal.ppat.1003607 PMC3771877 24068927

[22] Duplouy A, Hornett EA. Uncovering the hidden players in Lepidoptera biology: the heritable microbial endosymbionts. PeerJ. 2018;6:e4629. 10.7717/peerj.4629 PMC5947162 29761037

[23] Lo N, Casiraghi M, Salati E, Bazzocchi C, Bandi C. How many *Wolbachia *supergroups exist? Mol Biol Evol. 2002;19(3):341–6. 10.1093/oxfordjournals.molbev.a004087 11861893

[24] Duplouy A, Brattström O. *Wolbachia* in the genus *Bicyclus*: a forgotten player. Microb Ecol. 2018;75(1):255–63. 10.1007/s00248-017-1024-9 PMC5742604 28702705

[25] Shastry V, Bell KL, Buerkle CA, Fordyce JA, Forister ML, Gompert Z, et al. A continental-scale survey of *Wolbachia* infections in blue butterflies reveals evidence of interspecific transfer and invasion dynamics. G3 Genes Genom Genet. 2022;12(10):jkac213. 10.1093/g3journal/jkac213 PMC9526071 35976120

[26] Tagami Y, Miura K. Distribution and prevalence of *Wolbachia* in Japanese populations of Lepidoptera. Insect Mol Biol. 2004;13(4):359–64. 15271207 10.1111/j.0962-1075.2004.00492.x

[27] Chen X, Wang Z, Zhang C, Hu J, Lu Y, Zhou H, et al. Unraveling the complex evolutionary history of lepidopteran chromosomes through ancestral chromosome reconstruction and novel chromosome nomenclature. BMC Biol. 2023;21(1):265. 10.1186/s12915-023-01762-4 PMC10658929 37981687

[28] Li W, Cong Q, Shen J, Zhang J, Hallwachs W, Janzen DH, et al. Genomes of skipper butterflies reveal extensive convergence of wing patterns. Proc Natl Acad Sci USA. 2019;116(13):6232–7. 10.1073/pnas.1821304116 PMC6442542 30877254

[29] Zhang J, Cong Q, Grishin NV. Thirteen new species of butterflies (Lepidoptera: Hesperiidae) from Texas. Insecta Mundi. 2023;0969:1–58. PMC9880598 36713789

[30] Twort VG, Blande D, Duplouy A. One’s trash is someone else’s treasure: sequence read archives from Lepidoptera genomes provide material for genome reconstruction of their endosymbionts. BMC Microbiol. 2022;22(1):209. 10.1186/s12866-022-02602-1 PMC9426245 36042402

[31] Twort VG, Minet J, Wheat CW, Wahlberg N. Museomics of a rare taxon: placing Whalleyanidae in the Lepidoptera Tree of Life. Syst Entomol. 2021;46(4):926–37.

[32] Ribeiro P, Matos-Maraví P, Linke D, Meier J. The genome sequence of the Plain Longtail butterfly, *Spicauda simplicius* (Stoll, 1807) [version 1; peer review: 2 approved]. Wellcome Open Res. 2024;9:314. 10.12688/wellcomeopenres.22457.1 PMC11377922 39246520

[33] Andrews S. FastQC: a quality control tool for high throughput sequence data. 2010. Available online at: http://www.bioinformatics.babraham.ac.uk/projects/fastqc/.

[34] Ewels P, Magnusson M, Lundin S, Käller M. MultiQC: summarize analysis results for multiple tools and samples in a single report. Bioinformatics. 2016;32(19):3047–8. 10.1093/bioinformatics/btw354 PMC5039924 27312411

[35] Chen S, Zhou Y, Chen Y, Gu J. Fastp: an ultra-fast all-in-one FASTQ preprocessor. Bioinformatics. 2018;34(17):i884–90. 10.1093/bioinformatics/bty560 PMC6129281 30423086

[36] Bankevich A, Nurk S, Antipov D, Gurevich AA, Dvorkin M, Kulikov AS, et al. SPAdes: a new genome assembly algorithm and its applications to single-cell sequencing. J Comput Biol. 2012;19(5):455–77. 22506599 10.1089/cmb.2012.0021 PMC3342519

[37] Mikheenko A, Prjibelski A, Saveliev V, Antipov D, Gurevich A. Versatile genome assembly evaluation with QUAST-LG. Bioinformatics. 2018;34(13):i142–50. 10.1093/bioinformatics/bty266 PMC6022658 29949969

[38] Langmead B, Salzberg SL. Fast gapped-read alignment with Bowtie 2. Nat Methods. 2012;9(4):357–9. 10.1038/nmeth.1923 PMC3322381 22388286

[39] Danecek P, Bonfield JK, Liddle J, Marshall J, Ohan V, Pollard MO, et al. Twelve years of SAMtools and BCFtools. Gigascience. 2021;10(2):giab008. 10.1093/gigascience/giab008 PMC7931819 33590861

[40] Quinlan AR, Hall IM. BEDTools: a flexible suite of utilities for comparing genomic features. Bioinformatics. 2010;26(6):841–2. 10.1093/bioinformatics/btq033 PMC2832824 20110278

[41] Allio R, Schomaker-Bastos A, Romiguier J, Prosdocimi F, Nabholz B, Delsuc F. MitoFinder: efficient automated large-scale extraction of mitogenomic data in target enrichment phylogenomics. Mol Ecol Resour. 2020;20(4):892–905. 32243090 10.1111/1755-0998.13160 PMC7497042

[42] Li D, Liu CM, Luo R, Sadakane K, Lam TW. MEGAHIT: an ultra-fast single-node solution for large and complex metagenomics assembly via succinct de Bruijn graph. Bioinformatics. 2015;31(10):1674–6. 10.1093/bioinformatics/btv033 25609793

[43] Shen J, Cong Q, Borek D, Otwinowski Z, Grishin NV. Complete genome of *Achalarus lyciades*, the first representative of the Eudaminae subfamily of skippers. Curr Genomics. 2017;18(4):366–74. 10.2174/1389202918666170426113315 PMC5635620 29081692

[44] Katoh K, Standley DM. MAFFT multiple sequence alignment software version 7: improvements in performance and usability. Mol Biol Evol. 2013;30(4):772–80. 23329690 10.1093/molbev/mst010 PMC3603318

[45] Kearse M, Moir R, Wilson A, Stones-Havas S, Cheung M, Sturrock S, et al. Geneious Basic: an integrated and extendable desktop software platform for the organization and analysis of sequence data. Bioinformatics. 2012;28(12):1647–9. 22543367 10.1093/bioinformatics/bts199 PMC3371832

[46] Andermann T, Cano Á, Zizka A, Bacon C, Antonelli A. SECAPR - A bioinformatics pipeline for the rapid and user-friendly processing of targeted enriched Illumina sequences, from raw reads to alignments. PeerJ. 2018;6:e5175. 10.7717/peerj.5175 PMC6047508 30023140

[47] Ribeiro PG, Torres Jiménez MF, Andermann T, Antonelli A, Bacon CD, Matos-Maraví P. A bioinformatic platform to integrate target capture and whole genome sequences of various read depths for phylogenomics. Mol Ecol. 2021;30(23):6021–35. 34674330 10.1111/mec.16240 PMC9298010

[48] Espeland M, Breinholt J, Willmott KR, Warren AD, Vila R, Toussaint EFA, et al. A comprehensive and dated phylogenomic analysis of butterflies. Curr Biol. 2018;28(5):770–778.e5. 10.1016/j.cub.2018.01.061 29456146

[49] Wang X, Xiong X, Cao W, Zhang C, Werren JH, Wang X. Phylogenomic analysis of *Wolbachia* strains reveals patterns of genome evolution and recombination. Genome Biol Evol. 2020;12(12):2508–20. 33283864 10.1093/gbe/evaa219 PMC7719230

[50] Minh BQ, Schmidt HA, Chernomor O, Schrempf D, Woodhams MD, Von Haeseler A, et al. IQ-TREE 2: new models and efficient methods for phylogenetic inference in the genomic era. Mol Biol Evol. 2020;37(5):1530–4. 10.1093/molbev/msaa015 PMC7182206 32011700

[51] Lanfear R, Frandsen PB, Wright AM, Senfeld T, Calcott B. PartitionFinder 2: new methods for selecting partitioned models of evolution for molecular and morphological phylogenetic analyses. Mol Biol Evol. 2016;34(3):772–3. 10.1093/molbev/msw260 28013191

[52] Lanfear R, Calcott B, Kainer D, Mayer C, Stamatakis A. Selecting optimal partitioning schemes for phylogenomic datasets. BMC Evol Biol. 2014;14:82. 10.1186/1471-2148-14-82 PMC4012149 24742000

[53] Stamatakis A. RAxML version 8: a tool for phylogenetic analysis and post-analysis of large phylogenies. Bioinformatics. 2014;30(9):1312–3. 10.1093/bioinformatics/btu033 PMC3998144 24451623

[54] Hoang DT, Chernomor O, von Haeseler A, Minh BQ, Vinh LS. UFBoot2: improving the ultrafast bootstrap approximation. Mol Biol Evol. 2018;35(2):518–22. 10.1093/molbev/msx281 PMC5850222 29077904

[55] Altschul SF, Gish W, Miller W, Myers EW, Lipman DJ. Basic local alignment search tool. J Mol Biol. 1990;215(3):403–10. 2231712 10.1016/S0022-2836(05)80360-2

[56] Simão FA, Waterhouse RM, Ioannidis P, Kriventseva EV, Zdobnov EM. BUSCO: assessing genome assembly and annotation completeness with single-copy orthologs. Bioinformatics. 2015;31(19):3210–2. 26059717 10.1093/bioinformatics/btv351

[57] Valerio F, Twort VG, Duplouy A. A worked example of screening genomic material for the presence of *Wolbachia* infection. In: Fallon A, editor. *Wolbachia.* Methods in molecular biology. New York, NY: Humana; 2023. pp. 275–99. 10.1007/978-1-0716-3553-7_17 38006558

[58] Drummond AJ, Rambaut A, Shapiro B, Pybus OG. Bayesian coalescent inference of past population dynamics from molecular sequences. Mol Biol Evol. 2005;22(5):1185–92. 15703244 10.1093/molbev/msi103

[59] Bouckaert R, Vaughan TG, Barido-Sottani J, Duchêne S, Fourment M, Gavryushkina A, et al. BEAST 2.5: an advanced software platform for Bayesian evolutionary analysis. PLoS Comput Biol. 2019;15(4):e1006650. 10.1371/journal.pcbi.1006650 PMC6472827 30958812

[60] Heller R, Chikhi L, Siegismund HR. The confounding effect of population structure on Bayesian skyline plot inferences of demographic history. PLoS ONE. 2013;8(5):e62992. 10.1371/journal.pone.0062992 PMC3646956 23667558

[61] Darriba D, Taboada GL, Doallo R, Posada D. JModelTest 2: more models, new heuristics and parallel computing. Nat Methods. 2012;9(8):772. 10.1038/nmeth.2109 PMC4594756 22847109

[62] Rambaut A, Drummond AJ, Xie D, Baele G, Suchard MA. Posterior summarization in Bayesian phylogenetics using Tracer 1.7. Syst Biol. 2018;67(5):901–4. 10.1093/sysbio/syy032 PMC6101584 29718447

[63] Narita S, Nomura M, Kageyama D. Naturally occurring single and double infection with *Wolbachia* strains in the butterfly *Eurema hecabe*: transmission efficiencies and population density dynamics of each *Wolbachia* strain. FEMS Microbiol Ecol. 2007;61(2):235–45. 17506822 10.1111/j.1574-6941.2007.00333.x

[64] Reuter M, Keller L. High levels of multiple *Wolbachia* infection and recombination in the ant *Formica exsecta*. Mol Biol Evol. 2003;20(5):748–53. 12679529 10.1093/molbev/msg082

[65] Scholz M, Albanese D, Rota-Stabelli O, Donati C, Segata N. Large scale genome reconstructions illuminate *Wolbachia* evolution. Nat Commun. 2020;11(1):5235. 10.1038/s41467-020-19016-0 PMC7568565 33067437

[66] Wolfe TM, Bruzzese DJ, Klasson L, Corretto E, Lečić S, Stauffer C, et al. Comparative genome sequencing reveals insights into the dynamics of *Wolbachia* in native and invasive cherry fruit flies. Mol Ecol. 2021;30(23):6259–72. 33882628 10.1111/mec.15923 PMC9290052

[67] Keeling PJ. Horizontal gene transfer in eukaryotes: aligning theory with data. Nat Rev Genet. 2024; 25:416–30. 10.1038/s41576-023-00688-5 38263430

[68] Ellis EA, Storer CG, Kawahara AY. *De novo* genome assemblies of butterflies. Gigascience. 2021;10(6):giab041. 10.1093/gigascience/giab041 PMC8170690 34076242

[69] Hotaling S, Sproul JS, Heckenhauer J, Powell A, Larracuente AM, Pauls SU, et al. Long reads are revolutionizing 20 years of insect genome sequencing. Genome Biol Evol. 2021;13(8):evab138. 10.1093/gbe/evab138 PMC8358217 34152413

[70] Linke D, Hernandez Mejia J, Eche Navarro VNP, Salinas Sánchez L, Ribeiro PG, Elias M, Matos-Maraví P. Reduced palatability, fast flight, and tails: decoding the defence arsenal of Eudaminae skipper butterflies in a neotropical locality. J Evol Biol. 2024;37(9):1064–75. 39044333 10.1093/jeb/voae091

