# Supplementary figures and images for: Pervasive horizontal transmission of Wolbachia in natural populations of closely related and widespread tropical skipper butterflies
Source: BMC Microbiol. 2025 Jan 7;25:5. doi: 10.1186/s12866-024-03719-1 (PMC11706079; doi:10.1186/s12866-024-03719-1)

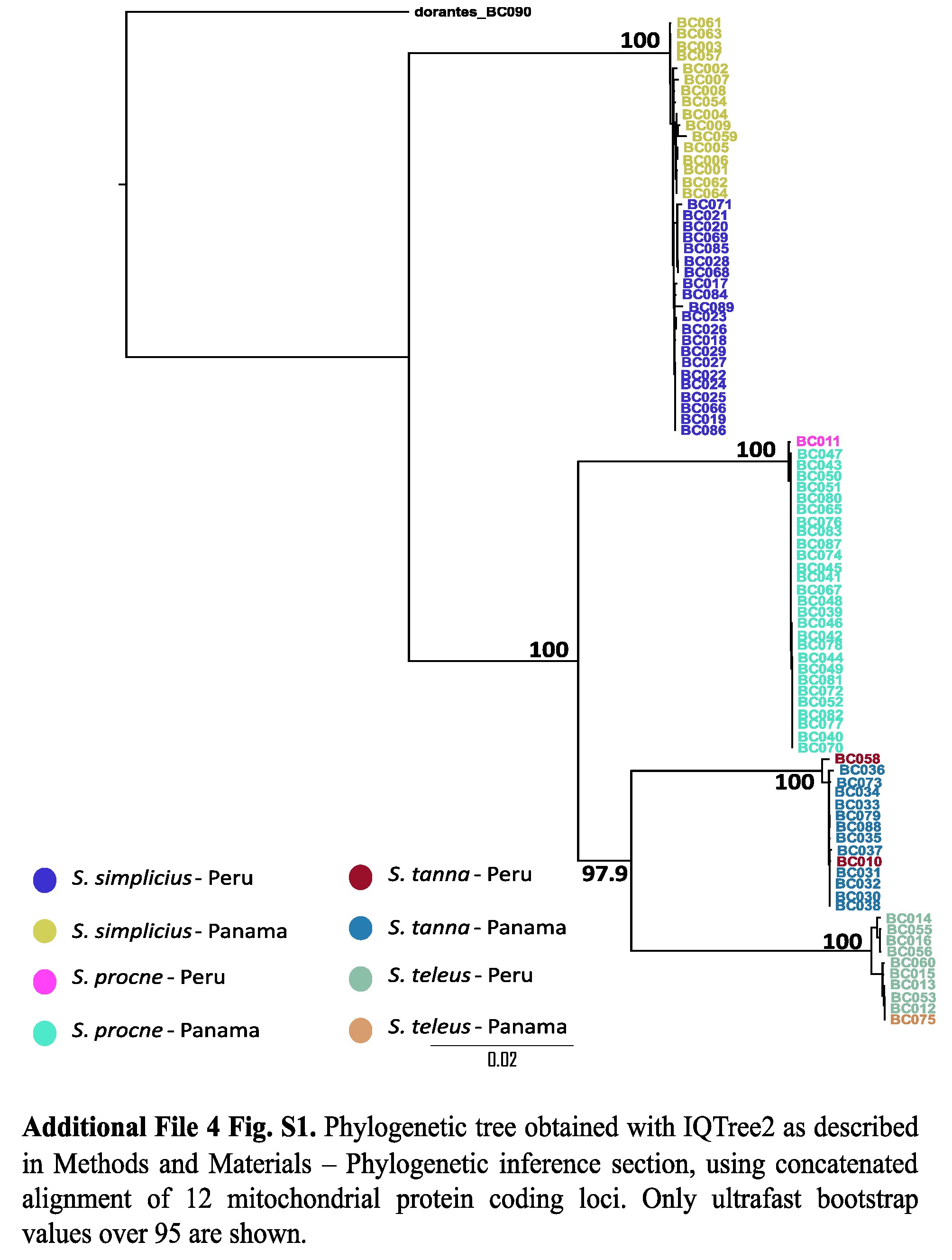

Supplement: Supplementary file 4 — Supplementary Material 4 [file 12866_2024_3719_MOESM4_ESM.jpg]

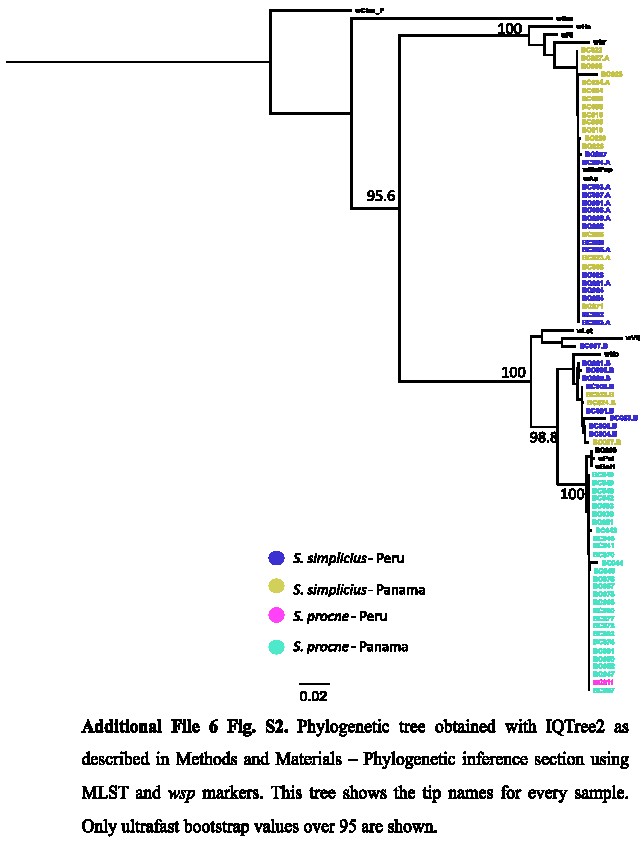

Supplement: Supplementary file 6 — Supplementary Material 6 [file 12866_2024_3719_MOESM6_ESM.jpg]
